# Non-Invasive Examination of Plant Surfaces by Opto-Electronic Means—Using Russet as a Prime Example

**DOI:** 10.3390/s16040452

**Published:** 2016-03-29

**Authors:** Matthias Klemm, Olga Röttger, Lutz Damerow, Michael Blanke

**Affiliations:** 1 INRES-Horticultural Science, Faculty of Agriculture, University of Bonn, D-53121 Bonn, Germany; m.klemm@gmx.net (M.K.); s7olroet@uni-bonn.de (O.R.); 2 Institute of Agricultural Engineering, Faculty of Agriculture, University of Bonn, D-53115 Bonn, Germany; damerow@uni-bonn.de

**Keywords:** 3D colour microscopy, glossiness, light reflection, luster sensor technology, russet, non-invasive technology, plant surface feature

## Abstract

(1) Background: Many disorders and diseases of agricultural produce change the physical features of surfaces of plant organs; in terms of russet, e.g., of apple or pear, affected fruit peel becomes rough and brown in color, which is associated with changes in light reflection; (2) Objective and Methods: The objective of the present project was an interdisciplinary approach between horticultural science and engineering to examine two new innovative technologies as to their suitability for the non-destructive determination of surfaces of plant organs, using russet as an example, and (a) an industrial luster sensor (type CZ-H72, Keyence, Japan) and (b) a new type of a three-dimensional (3D) color microscope (VHX 5000); (3) Results: In the case of russet, *i.e.*, suberinization of the fruit peel, peel roughness increased by *ca.* 2.5-fold from *ca.* 20 µm to *ca.* 50 µm on affected fruit sections when viewed at 200× magnification. Russeted peel showed significantly reduced luster, with smaller variation than russet-devoid peel with larger variation; (4) Conclusion: These results indicate that both sensors are suitable for biological material and their use for non-contact, non-invasive detection of surface disorders on agricultural produce such as russet may be a very powerful tool for many applications in agriculture and beyond in the future.

## 1. Introduction

Russeting is a relevant fruit quality parameter and its non-destructive detection a challenge worldwide, from California [1] to South Africa [2] and from China to Europe and Russia. The potential causes of russet on pome fruit include microclimate conditions as the prime environmental factor at the early stage of fruitlet development (30 days after full bloom in apple), such as persistent surface wetness as a result of humidity, rain or dew followed by intense sunshine, particular sprays including, e.g., copper formulations [3] employed for fungicidal effects, and the choice of variety and rootstock as prime genetic factors [4]. The physical properties of fruit peel affected by russet change and the peel becomes rough and brown in color compared with even, smooth and bright-colored unaffected peel [5,6].

In the past, the use of (a) colorimeters and (b) traditional gloss meters failed in russet detection [7]. The three candidates for russet detection of the change in physical properties are the loss of glossiness, decrease in brightness (L value in the CIELab scale) and emerging brown color. The detection of any brown skin defects such as scab, bruise injury or russet is hampered, because brown as a mixed color (red and blue) is not associated with any distinct peak at a particular wavelength in the light spectrum [7]; russet has long been a research objective without much success in applying non-invasive technologies [2]. This may be because the majority of traditional gloss meters require a large measuring area, a flat surface (e.g., steel or ceramics) and direct contact with the object, and often fail with use on biological material.

The work by Ward and Russinovitch (1986) [8] is a prime example of the difficulties experienced with only one type of glossmeter. To study the glossiness of fruit, authors had to carefully peel the bananas and place the detached peels on an absolutely even, flat surface for the measurement, where the glossmeter still touched the peel with subsequent browning; this indicates the challenge and shows that any non-destructive approach is a great milestone forward towards glossiness or shininess detection as part of the non-invasive surface examination of food and fruit.

Glossiness plays a relevant role as a quality feature of many agricultural products such as apple, cherry, plum, blueberry, eggplant and citrus fruit and often relates to its stage or ripeness, quality or visual appearance and possibly storability; several fruit are washed and then polished with, e.g., carnauba palm wax to extend their shelf life, reduce transpirational water loss and improve their visual appearance, including fruit such as, e.g., grapefruit, avocado, pineapple and apple. Many attempts have been made to measure and describe the glossiness of agricultural produce using qualitative and quantitative parameters, e.g., of glossy *versus* non-glossy or smooth *versus* rough. They either succeeded in detecting the ripeness stage destructively (e.g., Ward and Nussinovitch 1986) [8], or failed to detect the glossiness in a non-destructive way (e.g., Blanke *et al.*, 2001) [9]. Thereafter, several glossmeters failed to detect smoothness, glossiness or shininess on plant surfaces, but succeeded to detect the changes in light reflection associated with cherry fruit ripening [9].

The underlying physics of the optical detection of glossiness or smoothness or roughness of plant surfaces are as follows: Smooth surfaces reflect light beams at the same angle (a’) as that of the incoming angle (a), designated as directed light reflection. By contrast, rough, uneven surfaces result in diffuse light reflection, with the degree of diffuse reflections increasing proportionally with the degree of surface roughness. With diffuse reflection, light is reflected in all directions/angles upon incident light [10].

Microscopy has developed over the years from light microscopy (with a resolution of approximately 200 nm for conventional lenses) in the early days since the 1960s, to scanning (SEM) and transmission electron microscopy (TEM) in the 1980s with a resolution in excess of 0.1 nm, *i.e.*, greater than with SEM. All these techniques require considerable time and effort for sample preparation for SEM [11]) and TEM [12]); for high resolution photography, gold-coating of the specimen in a vacuum is beneficial. This was superseded by ESEM (Environmental Scanning Electron Microscopy) technology from *ca.* 2000, which allowed wet specimens to be examined, e.g., intact sensitive thin segment membranes, which retain the juice vesicles in the orange fruit [13]. In the meantime, energy dispersive X-ray analysis (EDX) became a further add-on to SEM, TEM and ESEM to enable the detection of certain elemental compositions such as K, Ca, Si, S and Cl on biological materials such as, e.g., grape berries [14] and on other plant surfaces, once the computing power became available and adapted for electron microscopy. The next milestone was the introduction of cryotechnique in *ca.* 1990, where the cryopreservation preserved the tissue, e.g., under examination both during gold-coating as well as during examination in the SEM. For agriculture, cryopreservation was a milestone, since tissue culture plays a major role, e.g., in rootstock propagation worldwide and cryopreservation prohibited tissue collapse (Blanke *et al.*, 1994) [15]. The micropropagated tissues are soft, grow in high humidity and are prone to instant dehydration. All the microscopic techniques were limited by destructive sampling, use of small specimen, ideal sample preparation including their vacuum-coating and two-dimensional examination and photography of surfaces in agriculture and beyond. To overcome this recognized limitation, the expertise was to tilt the specimen *ca.* 15° to create a pseudo–three dimensional (3D) impression (Blanke *et al.*, 1994) [15]. Fluorescence microscopy was developed, and it relies on a dye to highlight areas of particular interest; atomic force microscopy was also developed, and it can also be used for roughness detection. In 2013, the greatest milestone became the invention of non-destructive, 3D color microscopy of uncoated, non-dissected specimen, which now enables non-contact measurements, surfaces to be repeatedly examined and the undulations or surface roughness to be measured (Table 1).

Hence, the objective of this contribution was to investigate two new non-invasive technologies, a luster sensor and a new 3D color opto-electronic microscope, to determine their suitability to examine the change in fine structure and geometry and surface roughness using russet as an example.

## 2. Materials and Methods

### 2.1. Plant Material and Fruit Sourcing

Fully ripe, edible pears (*Pyrus domestica* L.) of cvs “Alexander Lucas” and “Abate Fetel” were locally sourced and examined for russet by an industrial luster sensor and 3D digital microscopy in autumn 2015.

### 2.2. Technique of Luster Measurement

The luster sensor selected for the study was of the new type CZ-H72 (Keyence Co., Osaka, Japan) and an amplifier type CZ V20 from the same company with a response time of less than 8 ms, which may be suitable for fruit sorting and grading. This type of sensor allows non-destructive surface measurements without close contact *viz.* without touching the specimen. The sensor is designed in a way that the specimen are 10–20 mm away from it with best results at 15 mm distance from the object, which results in an area of 5 mm for the observation; 3 mm area is an adjustable option (Table 2).

**Figure 1:**
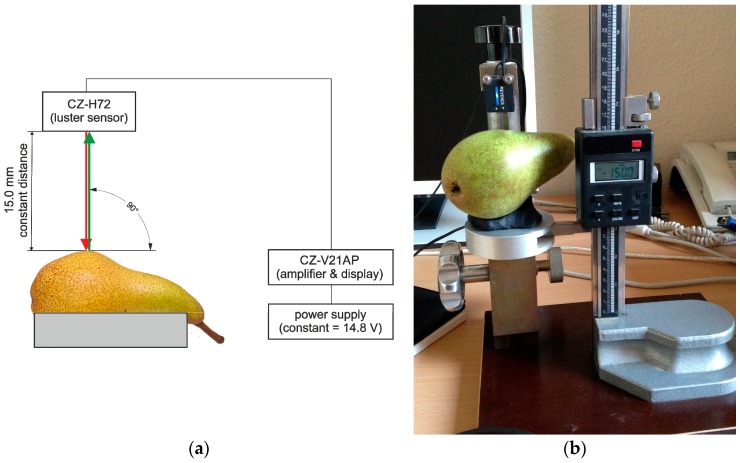
Measuring scheme (left, **a**) for the detection of physical surface features with the luster sensor showing the micro-manipulator on the right (**b**).

The luster sensor is a new model and was originally designed for industrial purposes such as package control; hence, the output values of the industrial sensor are luster levels without units. Consequently, we used a.u. (arbitrary units) in the diagrams to cater for this situation. Power was provided via an external, universal laboratory power supply at a constant 14.8 V (*ca.* 1 watt). We designed a micromanipulator to monitor and maintain the distance between the luster sensor and fruit surface rectangular and at a constant 15 mm with an area size of 5 mm (Figure 1).

### 2.3. Non-Invasive 3D Color Microscopy

**Figure 2:**
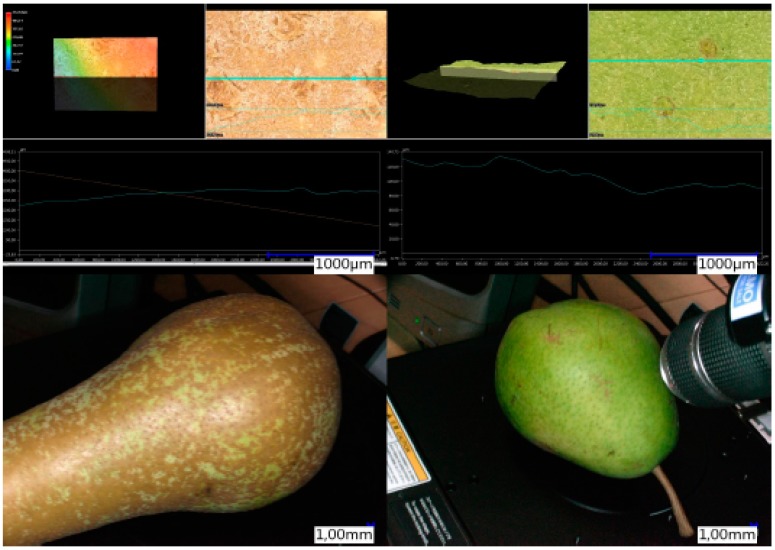
Determination of roughness using profile lines (blue) in the 3D microscope.

A new type of 3D color microscope type VHX 5000 (Keyence Co., Osaka, Japan) (Table 1) was employed for non-invasive examination of the roughness of intact specimen or intact fruit, *i.e.*, depth of peel surface undulations, without sectioning or the use of a vacuum-coated specimen. Profile lines (Figure 2) are used in the 3D images to determine the differences between the smallest and largest undulation and presented as roughness (Figure 2).

### 2.4. Data Processing

Eighteen pear fruits of each cultivar were examined for russeting; each pear fruit was measured 10 times for russet and russet-devoid areas. The luster values were statistically processed using RStudio with a significance level of 95% and graphically illustrated as Box-Whisker plots.

## 3. Results and Discussion

### 3.1. Fine Structure, Geometry and Roughness of Russet Peel

**Figure 3:**
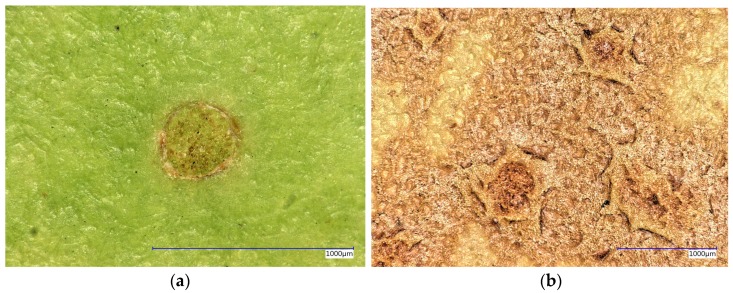
Color 3D micrographs of smooth-skinned, russet-devoid (**a**, left) and russet (**b**, right) pears at 200-fold magnification—the blue distance bar represents 1 mm.

The opto-electronic microscope type VHX 5000 revealed the fine structure of russet at 200-fold magnification in high resolution (2000 pixel) without sectioning (Figure 3).

The 3D microscope-enabled non-invasive measurements of the surface roughness, expressed as the depth of the undulations, on fresh plant material without sectioning, in contrast to traditional destructive microscopic examination. The three-dimensional examination of fresh fruit showed the geometry with the smooth appearance of the russet-devoid peel and the protuberant ridges on the russeted peel (Figure 3).

Russeting induced or increased the peel roughness by 2.5-fold from 20 µm with smooth-skinned, russet-devoid peel to 50 µm with russeted peel sections (Figure 2 and Figure 3).

### 3.2. Luster

**Figure 4:**
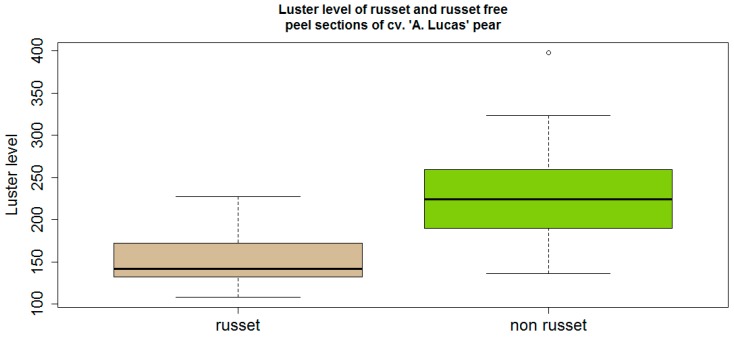
Significantly different luster level of russeted *versus* russet-devoid green peel sections of cv. “Alexander Lucas” (statistics: *p* = 3.3 × 10^−8^ << α = 0.05; *n* = 18).

The luster level of russeted peel of cv. “Alexander Lucas” ranged from a minimum of 108 to a maximum of 227 with a median of 140 (Figure 4, left). However, russet-devoid peel sections were associated with larger values, ranging from a minimum of 136 to a maximum of 340 with a median of 225 (Figure 4, right). Statistical analysis showed significant differences at 5% between russet and russet-devoid peel sections of the same cultivar.

**Figure 5:**
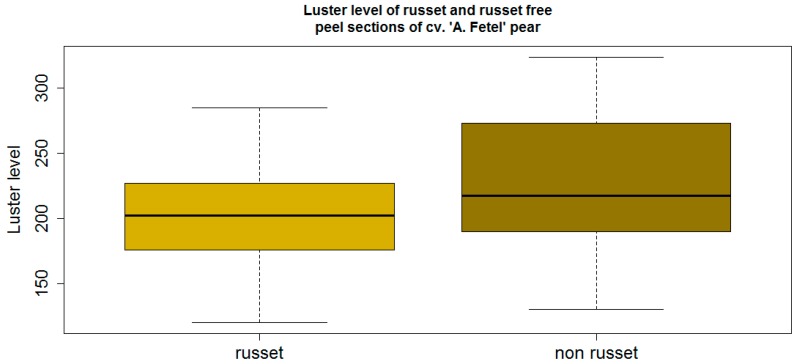
Luster values of russet and russet-devoid brown peel sections of cv. “Abate Fetel” pears (statistics: *p* = 0.09 >> α = 0.05; *n* =18).

The luster level of russet peel sections of cv. “Abate Fetel” ranged from a minimum of 120 and a maximum of 285 with a median of 202 (Figure 5, left). Russet-devoid peel sections showed similar values, ranging from a minimum of 130 to a maximum of 324 with a median of 217 (Figure 5, right). The lack of statistical significance at the 5% error rate in cv. “Abate Fetel” is attributed to the large variation of the russet-devoid peel, not the russeted peel, as shown in Figure 5.

### 3.3. Detection of Russet on Pome Fruits

The objective of the work was to investigate the surface properties of agricultural produce using russet as a particularly challenging example and two new non-invasive technologies with potential applications to food and fruit grading or sorting, a luster sensor and a new 3D color microscope, to examine the physical properties in fine structure and peel surface roughness at high resolution (2000 pixels). 

Russet has not been examined, to our knowledge, as to its physical properties such as luster features and regular light reflection. The luster sensor detected significant differences between russet and russet-devoid peel sections in cv. ”Alexander Lucas” and the same tendency, but without statistical significance, was found in cv. “Abate Fetel”. The examination by the luster sensor with one cultivar was a clearer discrimination of russet on the smoother-skinned cv. “Alexander Lucas” than on the less-smooth-skinned cv. “Abate Fetel”. This is due to more regular light reflection from russet-devoid, smooth-skinned fruit, but also due to larger variation than the russet values, which seemed more uniform.

## 4. Conclusions and Outlook

For the first time, an opto-electronic 3D color microscope provided an insight into the three-dimensional structure of a russeted fruit surface. The roughness, *i.e.*, depth of surface undulations, increased 2.5-fold from 20 µm in the smooth-skinned peel to 50 µm in the russet fruit sections.

The work has also shown that smooth-skinned, glossier fruit peel induced greater luster levels with unexpectedly larger variation, whereas rough and matt russeted peel reduced these values.

Questions always remain after this first study such as the effect of peel color and the ease of focusing. The results show the potential of the two non-invasive technologies for the examination and detection of russet on fruit, depending on cultivar, e.g., in fruit processing or on a grading or sorting line. These technologies may be of relevance to other surfaces of plant organs affected by russet, other peel disorders or diseases not only in pome fruit [16].

## Figures and Tables

**Table 1:** Comparison of the new microscopy features with traditional SEM/TEM.

	Traditional SEM/TEM	New Generation 3D Color Microscopy
Sample size	small, e.g., 1 cm × 1 cm	n.a.
Sample preparation	Large effort and timely	Not necessary
Sample (gold) coating in high vacuum	Often necessary for better results	Not necessary
Vacuum can lead to	Sample dehydration and tissue collapse	Specimen remains intact and unaffected
Non-invasive examination	Invasive	Non-invasive; allows repetition of the same samples
Examination	2D	3D
Microscope dimension	Occupies most of an air-conditioned room	Large but portable

**Table 2:** Technical features of the new CZ 72H luster sensor.

Feature	Value
LED source (red)	665 nm
Luster values	0 to 4000 a.u.
Spot size	3 or 5 mm diameter (optional)
Distance measurement	10–20 mm (center 15 mm)
Power	14.8 V (*ca.* 1 watt)
Response time	<8 ms
Weight	*ca.* 50 g
